# The ameliorative effect of pioglitazone and/or cod liver oil on hepatotoxicity induced by carbon tetrachloride in rats

**DOI:** 10.1186/s12263-025-00784-7

**Published:** 2025-12-01

**Authors:** Thoria Donia, Dina M. Elfeky, Sahar Saad El-Din Bessa, Ehab Tousson, Tarek M. Mohamed, Marian N. Gerges

**Affiliations:** 1 Biochemistry Division, Chemistry Department, Faculty of Science, Tanta University, Tanta, 31527 Egypt; 2 Medical Pharmacology, Faculty of Medicine, Tanta University, Tanta, Egypt; 3 Internal Medicine Department, Faculty of Medicine, Tanta University, Tanta, Egypt; 4 Zoology Department, Faculty of Science, Tanta University, Tanta, Egypt

**Keywords:** Hepatotoxicity, Hepatoprotective effects, Pioglitazone, Cod liver oil, Carbon tetrachloride, Adiponectin

## Abstract

Liver diseases rank among the foremost causes of mortality and morbidity worldwide. The PPARγ ligand, pioglitazone (PG), which has been clinically tested and is utilized in diabetes management, demonstrated a hepatoprotective effect in different studies. Furthermore, cod liver oil (COD) and its components exhibit a significant protective effect against hepatotoxicity in animals. This work aimed to investigate the impact of PG and/or COD on hepatotoxicity provoked by carbon tetrachloride (CCl_4_) in rats. Fifty albino Wistar rats were assigned to five groups (*n* = 10). Group I acted as a control. Group II received CCl_4_. Groups III and IV were administered with CCl_4_ and co-treated with oral PG or COD, respectively. Group V was administered with CCl_4_ and co-treated with both PG and COD. The duration of the experiment was 4 weeks. Liver function tests, hyaluronic acid (HA), and adiponectin levels were measured in serum. In liver tissue, adiponectin, tumor necrosis factor-alpha (TNF-α), and interleukin-10 (IL-10) gene expressions were evaluated by RT-PCR. Moreover, histopathological examinations and alpha-smooth muscle actin (α-SMA) immunoreactivity were assessed in hepatic tissues. Histopathological alterations were improved in the group treated with combined PG and COD, as indicated by amelioration of liver function tests, as well as decreased HA and elevated adiponectin levels in serum. In hepatic tissues, there was also an upregulation of adiponectin and IL-10 and a downregulation of TNF-α gene expression. Moreover, PG and COD decreased α-SMA expression in hepatic tissues as well. These findings indicate that PG and/or COD markedly mitigate liver damage caused by CCl_4_ in rats; however, further large-scale studies are necessary to prove this hypothesis.

**Graphical Abstract:**

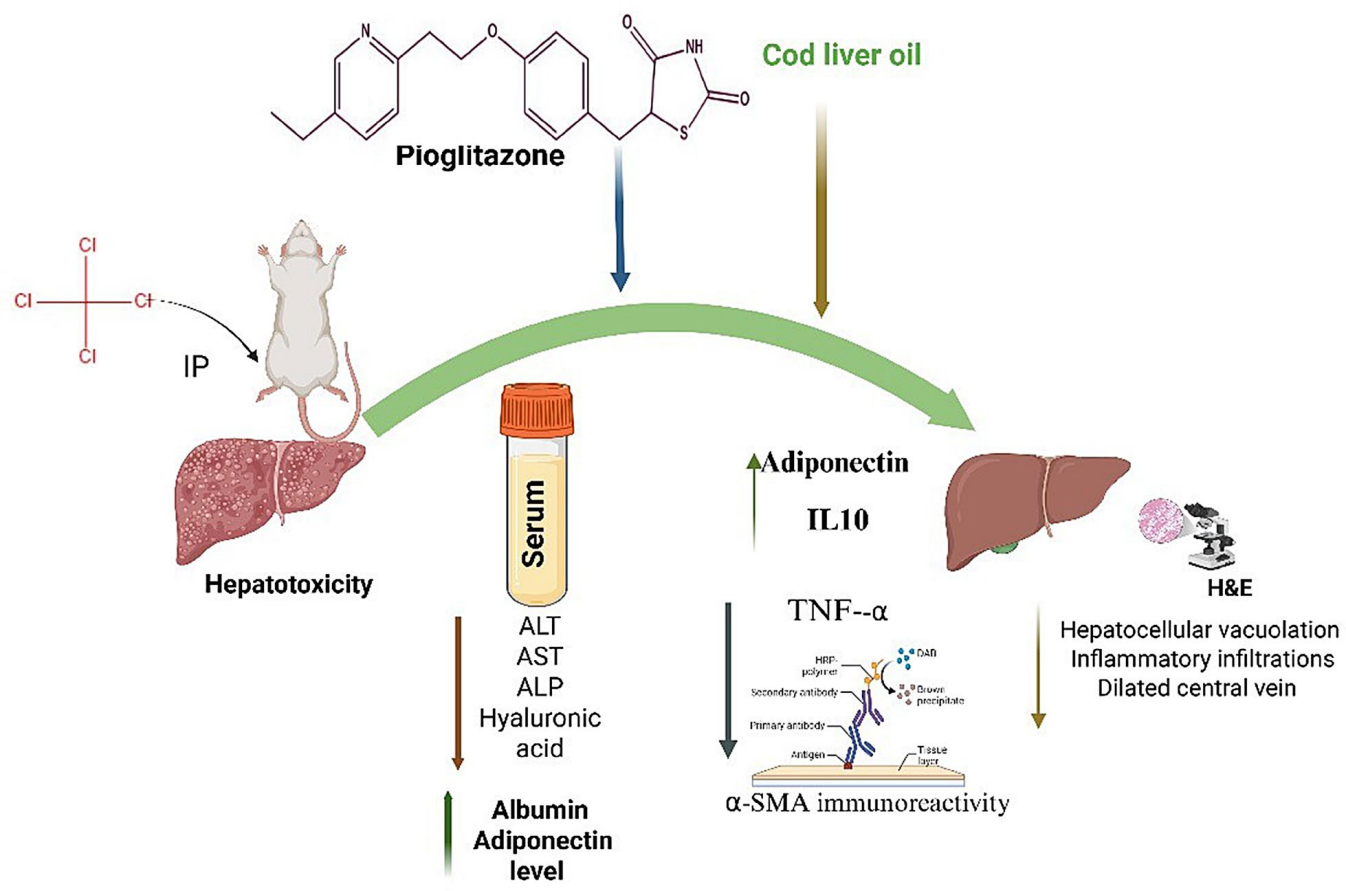

## Introduction

Liver diseases account for more than two million deaths each year, representing 4% of global mortality, or roughly one in every 25 deaths. The majority of deaths are attributable to complications of cirrhosis and hepatocellular carcinoma (HCC), with acute hepatitis accounting for a lesser percentage. The increase in mortality associated with cirrhosis is higher compared with other chronic conditions [1]. About 830,000 people died from liver cancer, which made up 8.3% of all cancer-related deaths worldwide [2].

Pathogenic microorganisms and viral hepatitis, hepatotoxins, drug overdose and duration, obesity and malnutrition, alcohol, autoimmune disorders, type-2 diabetes, and genetic factors have been proposed as probable risk factors for the development of liver diseases [3]. Liver diseases represent a broad range of conditions characterized by hepatocyte injury, inflammatory cell infiltration, and hepatic stellate cells (HSCs) activation, which together impair liver functions and interrupt its architecture [4]. Because of complicated etiology and scarcity of available treatments, liver diseases continue to pose a significant clinical practice challenge [5].

Interleukin-10 (IL-10) is a crucial anti-inflammatory cytokine with immunosuppressive properties, as it suppresses type-1 helper T cell (Th1) cytokines such as tumor necrosis factor-alpha (TNF-α) and interferon-γ (IFN-γ), which have been positively linked to increased necroinflammation and fibrosis [6, 7]. Evidence suggests that IL-10 modulates hepatic fibrogenesis as well [8]. IL-10 causes activated HSCs to senescence via the STAT3-p53 pathway, which suppresses liver fibrosis [9]. Additionally, IL-10 exhibited anti-inflammatory and antifibrotic influences in hepatitis C patients [10].

On the other hand, TNF-α is produced by macrophages, monocytes, Kupffer cells (KCs), and HSCs [11] with proinflammatory and cytotoxic effects [12]. TNF-α stimulates hepatocytes and KCs to produce transforming growth factor-beta (TGF-β), which in turn drives HSC activation, differentiation, proliferation, and survival. Furthermore, TNF-α promotes the stability of collagen and extracellular matrix (ECM) deposition [11].

Adiponectin is one of the most abundant adipocytokines that are formed and released into the bloodstream via white adipose tissue. It is chiefly involved in controlling glucose and lipid metabolism [13]. It also retains strong immune-modulatory, anti-inflammatory, and anti-fibrotic effects [14]. Adiponectin level is lowered in obesity and exhibits a negative correlation with insulin resistance, type 2 diabetes, and non-alcoholic fatty liver disease (NAFLD) [15, 16]. In addition, adiponectin knockout animals develop significant liver fibrosis after damage, whereas adiponectin gene delivery has the opposite outcome [17]. These actions are partially mediated by adiponectin via the AdipoR1 and AdipoR2 receptors, which are found in HSCs [18]. Adiponectin was found to attenuate HSCs’ proliferation and migration and induce their apoptotic death via triggering adenosine monophosphate-activated protein kinase (AMPK) and peroxisome proliferator-activated receptor-alpha (PPAR-α) [14].

Pioglitazone (PG) is a PPARγ ligand and one of the thiazolidinediones that is used to improve insulin resistance in type 2 diabetes [19]. In animals with NAFLD, it has been found to suppress hepatic steatosis and fibrosis [20, 21]. Additionally, PG successfully reduced the rise of serum liver enzymes and improved the degree of nonalcoholic steatohepatitis (NASH) in patients with NAFLD [22]. Moreover, it greatly lowered hepatic inflammation and insulin resistance in type 2 diabetic NAFLD patients [23]. Furthermore, PG and Ezetimibe combination improved liver histopathology and biochemistry in experimental metabolic dysfunction-associated steatohepatitis (MASH) models [24].

Cod liver oil (COD) is frequently taken as a nutritional supplement enriched with vitamins A and D as well as important omega-3 polyunsaturated fatty acids (PUFAs), including eicosapentaenoic acid (EPA) and docosahexaenoic acid (DHA) [25]. It was reported to suppress oxidative stress and inflammation. Additionally, COD was reported to lower cardio-metabolic risk factors, suppress chronic stress-induced cognitive impairment, treat osteomalacia and rickets, and exhibit antineoplastic effects [26–30]. Moreover, COD was found to ameliorate hepatic injury through several mechanisms, including the suppression of inflammatory cytokines and mediators of fibrosis, as well as apoptosis. It also exhibited a protective influence against hepatotoxicity by promoting the antioxidant defense system [31–33].

There is little published research on the biological effects of PG and COD against liver diseases, despite some indications of their hepatoprotective benefits. Also, no experimental studies have inspected the potential prevention of liver damage by PG in combination with COD. Therefore, this work was conducted to evaluate PG and/or COD effects on the progression of CCl_4_-prompted hepatotoxicity in rats via assessing gene expressions of adiponectin and both IL-10 and TNF-α.

## Materials and methods

### Chemicals

Pioglitazone was purchased from Arab Pharmaceutical Manufacturing Co., Ltd, Amman, Jordan. COD was obtained from Harraz Herbs shop, Cairo, Egypt. CCl_4_ (CAS Number: 56-23-5) was procured from Sigma Aldrich (USA). Additional chemicals were of superior analytical quality.

### Animals

Fifty male albino Wistar rats at 3 months old, weighing 120–150 g, were obtained from the National Research Center (Egypt) and conserved in plastic cages at 22 ± 1 °C temperature, 50 ± 5% relative humidity, and a 12-h dark/light cycle for 1 week to be acclimatized, having unrestricted access to food and water. In this context, they were maintained on a standard rodent diet composed of 20% casein, 15% corn oil, 55% corn starch, 5% salt mixture, and 5% vitaminized starch (Egyptian Company of Oils and Soap, Kafr-Elzayat, Egypt). The experimental design and study protocols followed the Faculty of Science’s Institutional Animal Care and Use Committee’s recommendations, Tanta University, Egypt (IACUC-SCI-TU-0488), and all methods are reported in accordance with ARRIVE guidelines (https://arriveguidelines.org).

### Experimental design

Fifty rats were distributed into five groups of ten each at random. Group I (Control): control rats. Group II (CCl_4_): rats received 3 ml/kg body weight of CCl_4_: olive oil (40: 60, v/v)/kg, by i.p. injection two times weekly [34]. Group III (CCl_4_ & PG): animals received oral PG (10 mg/kg body weight) suspended in distilled water every day using oral gavage [35]. Group IV (CCl_4_ & COD): rats were treated with COD (5 ml/kg body weight) orally daily by oral gavage [36]. Group V (CCl_4_ & PG & COD): rats were treated with both PG (10 mg/kg body weight) and COD (5 ml/kg body weight) orally daily. In addition, groups III, IV, and V were injected with CCl_4_: olive oil (40: 60, v/v)/kg twice weekly. The experiment lasted for four weeks.

After the experiment, animals were anesthetically sacrificed with halothane, and blood samples were taken from the rat’s abdominal aorta. The serum was collected and kept at -20 °C to be used for biochemical assays. The liver of rats was resected, washed with ice-cold isotonic saline, and separated into two parts; the first was fixed in 10% neutral formalin, and the second was stored at -80 °C until used for molecular analysis.

### Biochemical assays

Serum alanine aminotransferase (ALT), aspartate aminotransferase (AST), and alkaline phosphatase (ALP) activities were detected with (Biodiagnostic Co, Egypt) kits, and serum albumin level was measured using a commercial kit (Vitro Scient, Germany). Also, serum adiponectin was measured by Assay Max Rat adiponectin ELISA kit (ERA20500-1), and hyaluronic acid (HA) was determined using rat HA ELISA kit (MBS 704409) in line with the manufacturer’s instructions.

#### Quantitative RT-PCR analysis for adiponectin, IL-10, and TNF-α genes

From liver cells, total RNA was isolated utilizing the total RNA purification kit (Fermentas, #K0731, Thermo Fisher Scientific, USA). By absorption at 260 and 280 nm, both purity and concentration of RNA were detected employing Nanodrop (UV-VIS spectrophotometer Q5000, Quawell, USA). Revert Aid H Minus Reverse Transcriptase (Fermentas, #EP0451, Thermo Fisher Scientific, USA) was used to reverse transcribe 5 µg of total RNA into cDNA for each sample. The relative expression of the *IL-10*, *TNF-α*, *adiponectin*, and *β-actin* genes was assessed utilizing Step One Plus real-time PCR system (Applied Biosystems, USA) using 2X Maxima SYBR Green/ROX qPCR Master Mix (Thermo Scientific, # K0221, USA), and primers for these genes are represented in Table 1. For normalization, *β-actin* has been used as an internal reference. The fold change of relative gene expression was ascertained by the 2^−∆∆Ct^ method [37].

**Table 1:** Primer sequences used in qRT-PCR

Gene	Primers	Accession number	Amplicon length (bp)
*Adiponectin*	**F**: GGAGACGCAGGTGTTCTTGG **R**: AGCCCTACGCTGAATGCTGA	**NM_144744.3**	**152**
*IL-10*	**F**: CTTTCACTTGCCCTCATCC **R**: ACAAACAATACGCCATTCCC	**XM_006249712.5**	**265**
*TNF-α*	**F**: TTGCTTCTTCCCTGTTCC **R**: CTGGGCAGCGTTTATTCT	**AY427675.1**	**249**
*β-actin*	**F**: AAGTCCCTCACCCTCCCAAAAG **R**: AAGCAATGCTGTCACCTTCCC	**XM-032887061.1**	**96**

### Histopathological examination of liver tissue

Specimens were fixed, then dehydrated in a succession of increasing alcohol concentrations, underwent two xylene changes for clearing, and finally, paraffin wax was used to embed them. Ehrlich’s hematoxylin was used to stain liver sections, and eosin was used as a counterstain according to [38]. The remaining sections were kept in room temperature storage until being processed for immunohistochemistry. Histological scoring of liver damage was done on hematoxylin and eosin-stained sections. Vacuolated hepatocytes, cellular inflammatory infiltration, and dilated central vein were graded from 0 to 4 according to the modified Suzuki score. Scoring was done as follow: score 0 = no lesion, score 1 = minimal lesion involving less than 25% of liver tissue, score 2 = mild lesion involving 26%–50% of liver tissue, score 3 = moderate lesion involving 51%-75% of liver tissue and score 4 = severe lesions involving more than 76% of liver tissue (Table 2) [39].

**Table 2:** Suzuki’s score for grading the histological severity of hepatic damage

Modified histological score of Suzuki			
Score	Vacuolated hepatocyte	Dilated central vein	Inflammatory infiltration
0 (None)	Absent	Absent	Absent
1 Less than 25%	Minimal	Minimal	Minimal
2 (26% − 50%)	Mild	Mild	Mild
3 (51% − 75%)	Moderate	Moderate	Moderate
4 (More than 75%)	Severe	Severe	Severe

### Alpha-smooth muscle actin (α- SMA) immunoreactivity

For *α-SMA* immunoreactivity, the remaining paraffin-embedded hepatic sections were deparaffinized and hydrated. The activity of endogenous peroxidase was terminated by incubating for 5 min with 3% H_2_O_2_. The hepatic sections were kept overnight with *α-SMA* monoclonal antibody (Dako Corporation, Carpentaria, CA, USA) before being rinsed with phosphate buffer saline (PBS) for 5 min. After that, the monoclonal antibody was conjugated with a goat anti-mouse IgG antibody that had been biotinylated (Daco, LASB Universal Kit). Hematoxylin and eosin were used to counterstain sections [40]. When cells showed brown nuclear staining, it was assumed that they were positive for *α-SMA*; in contrast, negative nuclei had no staining and looked blue. Every stained slide was examined under the Olympus microscope. Quantitative calculation of α- SMA in 5 high power fields at 400 magnification of liver tissues was performed using ImageJ analysis software.

### Statistical analysis

The results are displayed as mean *±* SE. Utilizing GraphPad version 9, statistical analysis was performed. The statistical significance was distinct as *p* < 0.05.

## Results

### The impact of PG and/or COD on serum liver markers

In the CCl_4_ group, serum ALT, AST, and ALP activities were substantially higher (*p* < 0.05) than those of the control group. Additionally, CCl_4_ significantly reduced serum albumin levels (*p* < 0.05) in comparison to normal rats. In contrast, treating CCl_4_-administered animals with PG and/or COD exerted a marked decline in ALT, AST, and ALP activities (*p* < 0.05) in comparison to the CCl_4_ group. Interestingly, PG combined with COD ameliorated albumin levels to normal (Table 3).

Moreover, there was a substantial rise in serum HA levels in the CCl_4_ group (*p* < 0.05) when compared with control rats. Conversely, there was a marked drop in HA levels in the groups of CCl_4_-injected rodents administered with PG and/or COD (*p* < 0.05) in comparison with the CCl_4_ group. Furthermore, serum adiponectin levels showed a substantial decrease in the CCl_4_ group (*p* < 0.05) in comparison with normal rats, while the administration of PG alone and combined with COD exhibited a marked increase in serum adiponectin level (*p* < 0.05) when compared with the CCl_4_ group (Table 3).

**Table 3:** Serum biochemical parameters in different groups

	Control	CCl_4_	CCl_4_ & PG	CCl_4_ & COD	CCl_4_ & PG & COD
ALT (U/L)	29.7 ± 4.2	47.5 ± 0.23^a^	21.1 ± 2.14 ^b^	23.9 ± 0.7 ^b^	31.4 ± 2.8^b^
AST (U/L)	41.1 ± 3.7	80.6 ± 5.6^a^	30.5 ± 2.5^b^	58.4 ± 5.1^a, b,c^	56.4 ± 2.8^b, c^
ALP (U/L)	21.4 ± 2.2	38.4 ± 0.64^a^	21.8 ± 1.7^b^	34 ± 2.4^a, c^	36.7 ± 2.6^a, c^
Albumin (g/dl)	2.8 ± 0.09	2.22 ± 0.18^a^	2.75 ± 0.17	2.62 ± 0.17	2.81 ± 0.12^b^
Hyaluronic acid (ng/mL)	3.5 ± 0.92	16 ± 2.1^a^	3.3 ± 0.65^b^	5.8 ± 0.7^b^	9.8 ± 1.5^a, b,c^
Adiponectin (µg/mL)	41.3 ± 3.8	25.5 ± 1.8^a^	46.3 ± 4.8^b^	36.6 ± 0.97	45.8 ± 4.1^,b^

Data are mean ± SE (*n* = 8 in each group). ^a^*p*<0.05 recorded significance versus control, ^b^*p*< 0.05 recorded significance versus CCl_4_ group and ^c^*p*<0.05 recorded significance versus CCl_4_ & PG group. Data analysis was performed using the one-way ANOVA followed by the Tukey post hoc test. CCl_4_: Carbon tetrachloride, PG: Pioglitazone, COD: Cod liver oil

### The impact of PG and/or COD on the expression of *TNF-α*,* IL-10*, and *adiponectin* genes

**Fig. 1:**
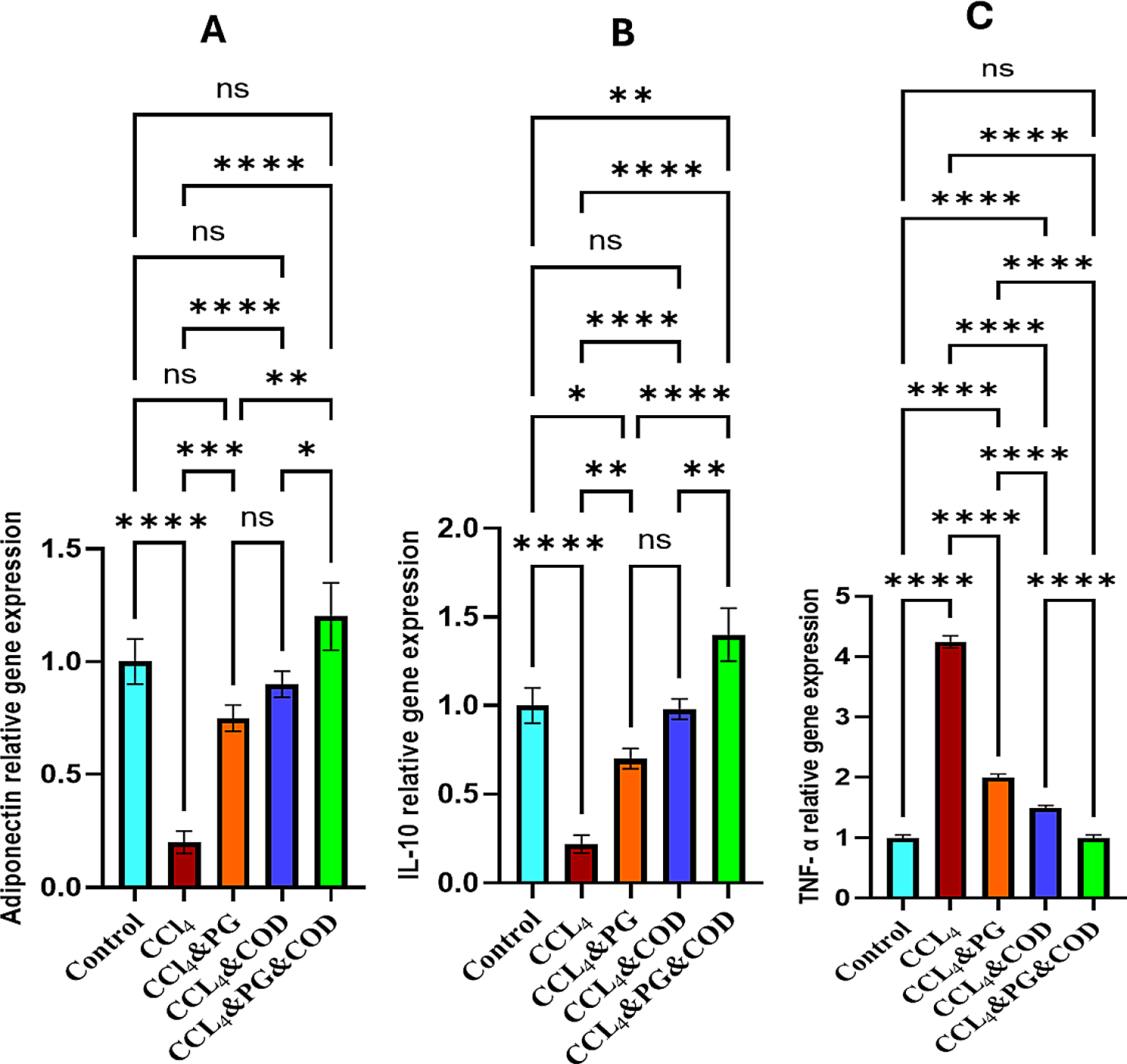
**(A-C)**: Gene expressions of adiponectin (**A**), IL-10 (**B**), and TNF-α (**C**) in rat liver using RT-PCR. Data are mean ± SE (*n* = 3). The expression level of each target gene in the control group was considered the baseline. Data analysis was performed using one-way ANOVA followed by Tukey post hoc test. *: *p* < 0.05, **: *p* < 0.01, ***: *p* < 0.001, and ****: *p* < 0.0001. CCl_4_: Carbon tetrachloride, PG: Pioglitazone, COD: Cod liver oil

The findings indicated a significant downregulation of the anti-inflammatory genes, *adiponectin and IL-10* expressions in the rats that received CCl_4_ alone (*P* < 0.05) as compared to control group. On the contrary, the mRNA expressions of *adiponectin* and *IL-10* were significantly upregulated following administration of PG and/or COD (*P* < 0.05) in comparison with the CCl_4_-injected animals, with the maximum results in the combination group (Fig. 1A, B).

On the other hand, the pro-inflammatory gene, *TNF-α*, expression was notably upregulated in the CCl_4_ group (*P* < 0.05) when compared with control group. However, its expression was suppressed following receiving PG and/or COD (*P* < 0.05) in comparison to CCl_4_-injected animals, with the lowest result in the combined treatment (Fig. 1C).

### Histopathological examination

**Fig. 2:**
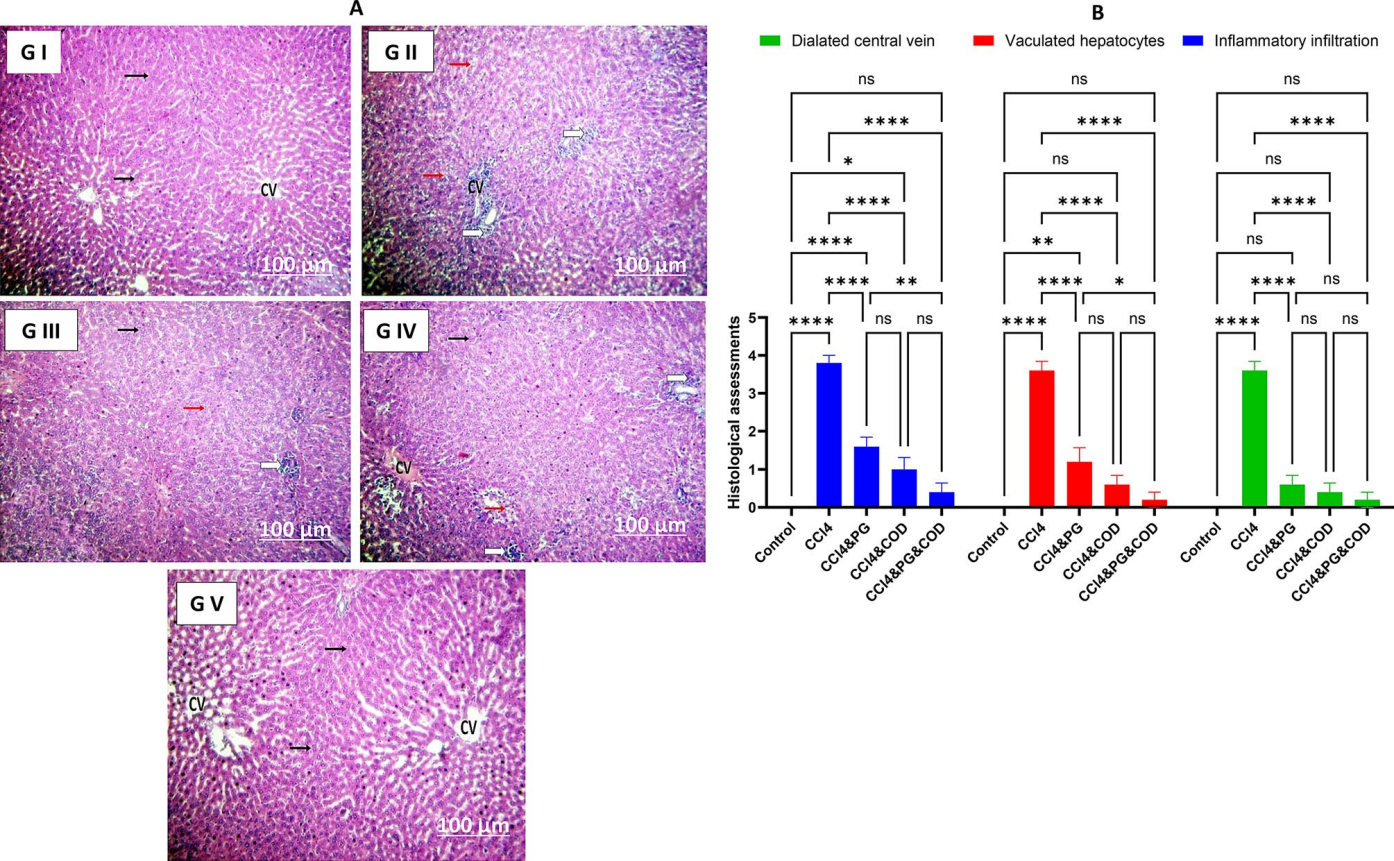
(**A**) Photomicrographs of rats’ liver sections in the different experimental groups stained with Haematoxylin & Eosin. **GI**: Liver sections in the control group revealed normal structure of hepatocytes (Hp) (Black arrows) and central vein (cv). **G II**: The liver section of the CCl_4_ group showed severe inflammatory infiltration (White arrows), hepatic vacuolation (Red arrows), and dilated central vein (CV). **G III**: Liver sections of co-treated rats with CCl_4_ and PG showed moderate injury with moderate vacuolation (Red arrow), beside mild inflammatory infiltration (White arrow), and dilated central vein. **G IV**: Liver section in rats co-treated with CCl_4_ and COD exhibited mild hepatocellular vacuolation (Red arrow) with minimal inflammatory infiltration (White arrow) and dilated central vein. **G V**: Liver sections in rats co-treated with CCl_4_, PG, and COD showed a very good degree of improvement, as shown by normal hepatocytes (Black arrows). CCl_4_: Carbon tetrachloride, PG: Pioglitazone, COD: Cod liver oil. (**B**) Suzuki’s score results in the experimental groups. Data are mean ± SE (*n* = 3). Data analysis was performed using one-way ANOVA followed by Tukey post hoc test. *: *p* < 0.05, **: *p* < 0.01, and ****: *p* < 0.0001. CCl_4_: Carbon tetrachloride, PG: Pioglitazone, COD: Cod liver oil

The histopathological investigations of liver sections of all experimental groups were revealed in Fig. 2A. Liver sections of the control rats exhibited normal histological features with normal liver cells and central vein **(G I)**. In the CCl_4_ group, liver sections showed severe cellular infiltration, hepatocellular vacuolation and marked dilated central vein **(G II**). Additionally, liver sections of rodents administered with CCl_4_ and PG revealed a substantial reduction in the impairment with moderate hepatic vacuolation as well as mild inflammatory infiltration and dilated central vein **(G III)**. Moreover, liver sections of animals administered with CCl_4_ and COD exhibited mild hepatocellular vacuolation with minimal inflammatory infiltration and dilated central vein **(G IV)**. Furthermore, liver sections of animals received CCl_4_, PG, and COD showed a very good degree of improvement of histopathological alterations **(G V)**. Results of Suzuki’s score in the investigated groups were presented in (Fig. 2B).

### α-SMA expressions

**Fig. 3:**
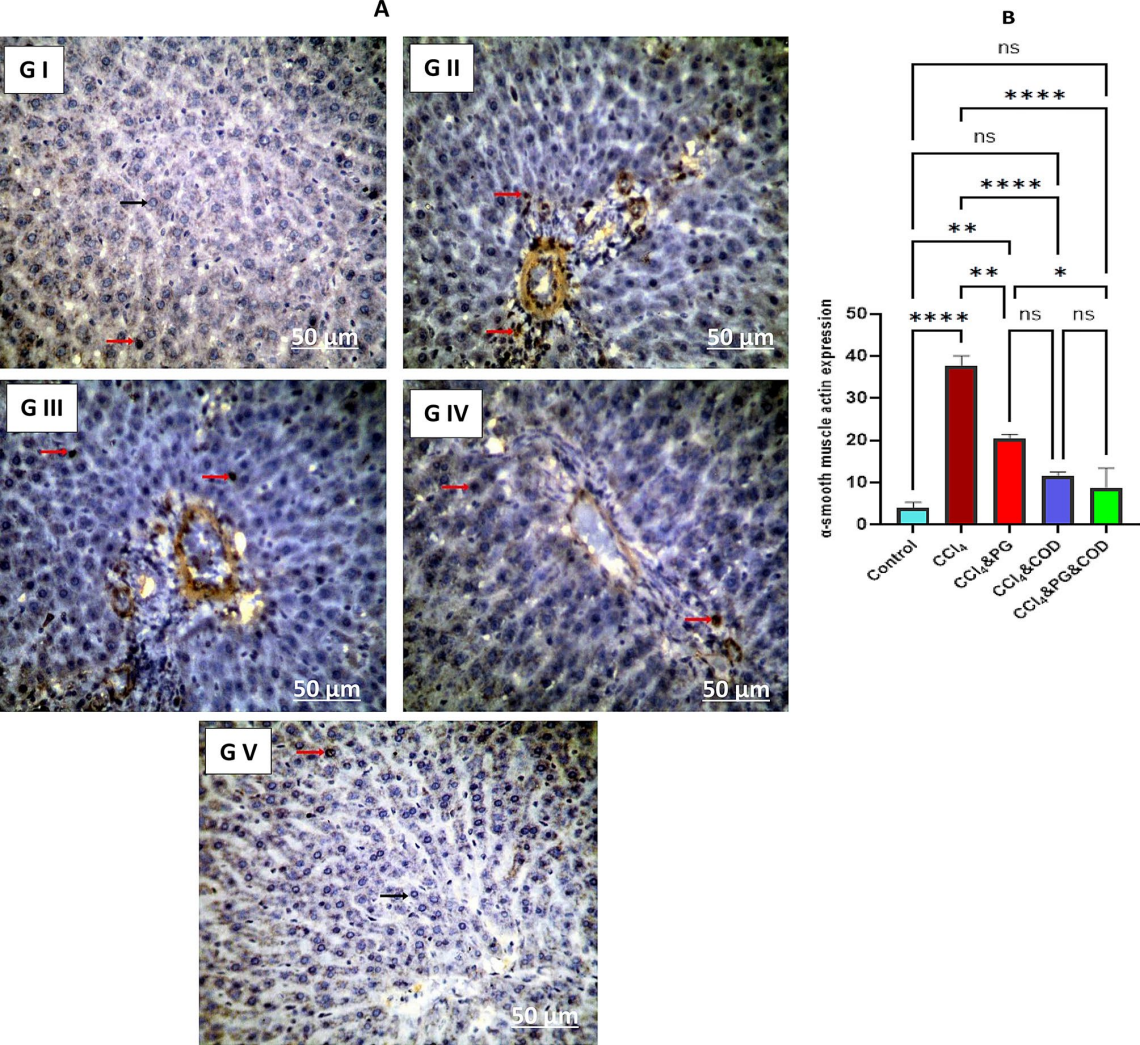
(**A**) α-SMA expressions in the rat liver of all groups. **G I**: The liver section of control group revealed negative expressions for α-SMA (Black arrow). **G II**: Liver section of CCl_4_ group showed strong positive α-SMA expressions (Red arrows). **G III**: The liver section of co-treated rats with CCl_4_ and PG shows moderate positive reactions for α-SMA expressions (Red arrow). **G IV**: The liver section of co-treated rats with CCl_4_ and COD shows mild positive reactions for α-SMA expressions (Red arrows). **G V**: Liver section of co-treated rats with CCl_4_, PG, and COD shows faint or negative reaction for α-SMA expressions (Black arrows) in hepatocytes. CCl_4_: Carbon tetrachloride, PG: Pioglitazone, COD: Cod liver oil. (**B**) α-smooth muscle actin expression in the experimental groups. Data are mean ± SE (*n* = 3). Data analysis was performed using one-way ANOVA followed by Tukey post hoc test. *: *p* < 0.05, **: *p* < 0.01, and ****: *p* < 0.0001. CCl_4_: Carbon tetrachloride, PG: Pioglitazone, COD: Cod liver oil

The expression of α-SMA in control and treated groups was displayed in Fig. 3A. Liver sections in the control group showed negative expressions for α-SMA, evidenced by blue stains because of normal liver cells and central hepatic vein **(G I)**. The hepatic sections of the CCl_4_ group showed strong positive reactions for α-SMA expressions **(G II)**. In contrast, liver sections of rats co-treated with CCl_4_ and PG showed moderate positive reactions for α-SMA expressions **(G III)**. Additionally, liver sections of rats received CCl_4,_ and COD showed mild positive reactions for α-SMA **(G IV)**. Moreover, liver sections of rats treated with CCl_4_, PG and COD showed faint or negative reaction for α-SMA in hepatocytes **(G V)**. Quantitative calculation of α-SMA in hepatic tissues was presented in Fig. 3B.

## Discussion

This study demonstrated that treatment with PG and COD ameliorated CCl_4_-induced toxicity, leading to the normalization of serum hepatic enzymes (like AST, ALT, and ALP) and albumin, as well as decreased HA and elevated adiponectin levels in serum. In hepatic tissues, there was also an upregulation of adiponectin and IL-10 and a downregulation of TNF-α gene expression. Moreover, PG and COD decreased α-SMA expression in hepatic tissues as well, which was further confirmed by histological findings.

The liver parenchyma is frequently damaged, leading to hepatocellular injury due to the production of free radicals during the metabolism and elimination of xenobiotic compounds. Various hepatotoxic chemicals, such as paracetamol, dimethyl nitrosamine, D-galactosamine/LPS, CCl_4_, along with alcoholism and viral infections, have been linked to this type of damage [41]. Treating hepatotoxicity with existing pharmaceuticals is challenging due to their adverse effects and intrinsic toxicities. Consequently, there is a necessity to devise an effective alternative for the management of liver treatment that ensures both efficacy and safety. The hepatoprotective agent must possess the capability to reinstate the liver’s normal architecture and maintain the physiological systems disrupted by hepatotoxins [42]. Therefore, we tested PG or/and COD for their protective effects in a hepatic toxicity model.

Carbon tetrachloride (CCl_4_) is an efficient hepatotoxin that harms the liver in animal models. The activation of several cytochromes, such as CYP2E1, CYP2B1, CYP2B2, and potentially CYP3A, which generate the trichloromethyl radical (CCl_3_*), is the cause of this toxicity. CCl_3_* can bind to biological molecules such as proteins, nucleic acids, and lipids, which hinders lipid metabolism, resulting in fatty degeneration. Additionally, it can start hepatic carcinogenesis by combining with DNA. Moreover, lipid peroxidation and membrane damage can result from CCl_3_*’s generation of more reactive CCl_3_OO* when exposed to oxygen [43]. Hence, this work studies the potential hepatoprotective properties of PG and COD against CCl_4_-prompted toxicity in rats.

In this study, a significant increase in serum ALT, AST, and ALP activities was observed in CCl_4_-injected animals compared to control rats, which is in agreement with previous studies [44, 45]. The results may be attributed to the ability of CCl_4_ metabolites to induce lipoperoxidation of the plasma membrane, initiating a cascade that leads to the degradation of cellular membrane integrity and the efflux of enzymes into the extracellular environment [46]. On the other hand, serum ALT, AST, and ALP activities significantly decreased in all treated rat groups compared to the CCl_4_ group. Consistent with our findings, PG was observed to diminish serum ALT and AST activities in rats administered CCl_4_ [47]. PG also demonstrated a reduction in serum transaminase activities in patients with NAFLD and NASH [23, 48, 49]. A prior study also demonstrated the hepatoprotective effects of COD, evidenced by a notable reduction in elevated serum liver enzymes and the preservation of normal hepatocyte morphology [36, 50]. Furthermore, COD was observed to reduce the elevation of serum ALT and AST activities and alleviate oxidative stress in the hepatic tissues of rats administered with CCl_4_ [51]. Consequently, these findings suggest that PG and/or COD therapy prevents the CCl_4_-induced deterioration of functional integrity in the hepatocyte membrane and the efflux of sensitive enzymes.

In this work, we observed a significant reduction in serum albumin levels in the CCl_4_ group compared to the control group. This may be due to the impairment of the liver’s ability to synthesize albumin [52]. Notably, PG and COD maintained serum albumin levels within the normal range. The results may be attributed to the hepatoprotective effects of both PG and COD. These findings align with a previous study, which indicated that PG pre-treatment prohibited the lipopolysaccharide (LPS)-triggered decline in serum albumin in rats [53]. COD also mitigated the reduction in serum albumin levels induced by sodium nitrite in rats [50].

Hyaluronic acid is an essential ECM component and is regarded as the benchmark for evaluating liver damage and fibrosis [54]. In our study, HA levels demonstrated a significant increase in the CCl_4_-treated animals compared to control rats, aligning with previous research [55]. Given that hepatic stellate cells (HSCs) participate in HA synthesis in the liver, their activation and proliferation during fibrogenesis would enhance HA expression, along with other connective tissue constituents. The majority of newly synthesized HA is discharged into the extracellular space, with a fraction potentially entering the bloodstream, a phenomenon exacerbated by liver necrosis. Furthermore, hepatic sinusoidal endothelial cells degrade nearly 90% of HA in the bloodstream. Fibrosis, however, hinders the functionality of sinusoidal endothelial cells. The increase in serum HA levels in the CCl_4_ group can be attributed to its leakage into circulation and a reduced degradation rate resulting from the dysfunction of sinusoidal endothelial cells [56]. This study demonstrated that serum HA levels significantly decreased in groups treated with PG and/or COD compared to the CCl_4_ group, indicating their capacity to mitigate CCl_4_-induced hepatotoxicity. These observations align with previous research indicating that PG significantly reduced serum HA levels in CCl_4_-treated and NASH rats [57, 58]. The impact of COD on decreasing serum HA levels may be elucidated by the presence of omega-3 polyunsaturated fatty acids [59].

The pro-inflammatory cytokine TNF-α serves as a principal endogenous mediator of hepatic toxicity [60]. The present study indicates that TNF-α expression in liver tissues was elevated in the CCl_4_ group, corroborating previous research findings [61, 62]. These findings may result from CCl_4_’s capacity to activate cytoplasmic NF-κB, which subsequently translocates to the nucleus and initiates TGF-β gene expression. TGF-β enhances collagen deposition between cells, stimulating HSCs and transforming them into myofibroblasts. Furthermore, NF-κB stimulates the transcription of TNF-α, initiating the upregulation of specific interleukins and other inflammatory mediators through interactions within the inflammatory cascade [63]. Nonetheless, TNF-α gene expression was markedly downregulated by PG and/or COD administration relative to CCl_4_. These findings align with previous studies demonstrating that PG mitigates liver damage induced by ethanol and LPS through the inhibition of TNF-α expression [64]. PG also reduced hepatic TNF-α expression in NASH rats [65]. PPAR-γ, a ligand-activated transcription factor within the nuclear hormone receptor superfamily, is regarded as a vital metabolic regulator of hepatic lipid metabolism and inflammation [66]. PPARγ exerts an anti-inflammatory effect by inhibiting the production of inflammatory cytokines, such as TNF-α [67]. Hence, PG may decrease TNF-α expression in the liver of CCl_4_-treated rats by activating PPARγ. Furthermore, COD was reported to reduce sodium nitrite-induced hepatocyte injury in rats by decreasing TNF-α levels [36]. COD contains omega-3 fatty acids, which have been shown to support anti-inflammatory responses [68].

Interleukin-10 gene therapy demonstrated the ability to mitigate CCl_4_-induced hepatotoxicity in animals by limiting inflammation, reducing collagen deposition, and facilitating ECM degradation [9]. Our findings demonstrated a notable downregulation of IL-10 gene expression in hepatic tissues post CCl_4_ administration relative to the control rats, corroborating previous research [69, 70]. The mRNA expression of IL-10 was significantly elevated after the administration of PG and/or COD compared to the CCl_4_ group, with the most pronounced increase observed following the combined administration of both PG and COD. A previous study demonstrated that PG increased hepatic IL-10 expression in mice subjected to a high-fat diet, attributable to its role as a PPARγ agonist [71].

Furthermore, adiponectin has been shown to downregulate the expression of TGF-ß1, NF-κB, TNF-α, and α-SMA, thereby diminishing HSC activation and inhibiting hepatotoxicity [72, 73]. Furthermore, in adiponectin-knockout mice, the administration of CCl_4_ induced more extensive liver fibrosis compared to wild-type mice, whereas the prior administration of an adenovirus that produces adiponectin mitigated liver fibrosis following CCl_4_ injection [74]. Additionally, the adiponectin-mimetic peptide analogue ADP355 was shown to eliminate hepatic fibrosis in mice exposed to CCl_4_ [75]. The current study revealed a significant reduction in serum adiponectin levels and adiponectin expression in hepatic cells within the CCl_4_ group, corroborating prior research findings [76, 77]. Serum adiponectin levels and hepatic mRNA expression were significantly increased in rats treated with PG and/or COD compared to the CCl_4_ group, with the most pronounced upregulation observed following the combined administration of PG and COD. These findings align with another study indicating that PG elevated plasma adiponectin levels, which have anti-inflammatory and antifibrotic effects on NAFLD [78]. Furthermore, it improved liver fibrosis by enhancing adiponectin expression [79]. Additionally, the impact of COD on increasing adiponectin expression may be linked to the presence of omega-3 fatty acids [80]. Consequently, adiponectin may reduce hepatic inflammation and fibrosis, indicating a potential clinical application for adiponectin and its agonists in the treatment of liver diseases.

The increase in α-SMA levels is a well-documented consequence of HSC activation. Upon activation, stellate cells transform into myofibroblasts, which initiate the production and secretion of ECM proteins, thereby promoting the progression of fibrosis [81]. TNF-α was identified as a pivotal cytokine for enhancing α-SMA gene expression [82]. Moreover, α-SMA expression was increased in the hepatic tissues of mice deficient in IL-10 [83]. Adiponectin can induce apoptosis in HSCs, resulting in the depletion of α-SMA proteins in these cells [84]. Here, the administration of CCl_4_ significantly increased the α-SMA-positive area and expression, consistent with previous studies [85–87]. In contrast, treatment with PG and/or COD significantly reduces the α-SMA-positive area and its expression, likely attributable to their capacity to downregulate TNF-α and upregulate both IL-10 and adiponectin in hepatic tissues. These observations correspond with prior studies demonstrating that PG reduced α-SMA expression in liver fibrosis models [58, 88]. These findings indicate that PG and COD may reduce HSC activation during the pathogenesis of CCl_4_-induced hepatic fibrosis.

The histopathological examination revealed that CCl_4_ administration induced significant pathological changes, including inflammatory infiltration, vacuolation, and dilation of the central vein, consistent with previous studies [69, 89]. Following the administration of PG and/or COD, there was a significant restoration of hepatic damage caused by CCl_4_, characterized by a distinctly normal hepatocyte architecture, corroborating previous research [47]. Histopathological changes were lessened when PG was administered for two weeks following CCl_4_ treatment, according to another research [90]. Furthermore, COD was observed to mitigate histopathological alterations caused by hepatotoxins, including sodium nitrite and isoniazid-rifampin [50, 91].

## Conclusion

The current study demonstrates that both PG and COD, when administered individually or in combination, exhibit protective effects against CCl_4_-induced liver injury in rats. Treatment with PG and/or COD significantly improved liver function, reduced serum HA, and increased adiponectin levels. At the molecular level, PG and/or COD enhanced anti-inflammatory (IL-10, adiponectin) and suppressed pro-inflammatory (TNF-α) markers. PG and/or COD also reduced α-SMA expression, indicating attenuation of HSCs activation. Histological findings further supported the biochemical and molecular outcomes, particularly in the group receiving the combined PG and COD treatment. These results suggest that PG and COD, especially when used together, may serve as effective therapeutic agents against liver diseases. While the study examines the protective effects of PG and/or COD against CCl_4_-induced liver toxicity in male rats, its applicability to female rats and in other liver conditions such as fatty liver disease, cirrhosis, and hepatocellular carcinoma remains uncertain. Additionally, there is a need for the deep exploration of the hepatoprotective mechanism of PG on CCl_4_-induced hepatotoxicity in future studies. Also, the effects of PG and/or COD on liver hydroxyproline content, α-SMA protein and mRNA expression, and oxidative stress markers require further investigation. Given the limited research available, further studies using genetically modified animals that overexpress adiponectin are needed to better understand the long-term effects of adiponectin modulation on liver diseases.

## Data Availability

All data generated or analyzed during this study are included in this manuscript.
